# Mast Cell Repopulating Ability Is Lost During the Transition From Pre-HSC to FL HSC

**DOI:** 10.3389/fimmu.2022.896396

**Published:** 2022-07-08

**Authors:** Momoko Yoshimoto, Astrid Kosters, Samuel Cornelius, Noemi Valiente, Haizi Cheng, Augusto Latorre, Chika Nishida, Eliver E. B. Ghosn, Michihiro Kobayashi

**Affiliations:** ^1^ University of Texas Health Science Center at Houston, Center for Stem Cell and Regenerative Medicine, Houston, TX, United States; ^2^ Lowance Center for Human Immunology, Division of Immunology and Rheumatology, Department of Medicine and Pediatrics, Emory University School of Medicine, Atlanta, GA, United States; ^3^ Emory Vaccine Center, Yerkes National Primate Research Center, Emory University School of Medicine, Atlanta, GA, United States

**Keywords:** HSC, mast cell (MC), fate-mapping, fetal liver, hemogenic endothelial cells

## Abstract

Recent advances in developmental immunology have revealed a hematopoietic stem cell (HSC)-independent origin for various innate immune lineages, including mast cells (MCs). It is now established that adult bone marrow (BM) long-term HSCs do not regenerate MCs but, instead, the physiological production of MCs starts before the emergence of HSCs in the aorta-gonad-mesonephros (AGM) region and is mostly completed before birth. However, while the AGM region represents a major site of MC generation during ontogeny, whether the first emerging HSCs in the AGM or fetal liver (FL) possess the potential to regenerate MCs is unknown. Here, we combined three fate-mapping mouse models with detailed HSC transplantation assays to determine the potential of AGM and FL HSCs to produce MCs. We show that HSCs from E11.5 AGM and E12.5 FL efficiently repopulated MCs in recipients. In stark contrast, HSCs from ≥E14.5 FL failed to reconstitute MCs. An Endothelial (EC) fate-mapping study confirmed the EC origin of the majority of MCs. Additionally, our HSC-labeling showed that HSCs do not produce MCs in a physiological setting. Hence, although most MCs are generated and maintained *via* an HSC-independent pathway, the earliest HSCs to emerge in the AGM and seed the early FL can produce MCs, but only during a minimal time window. Our results challenge the stem cell theory in hematology and EC-derived mast cells may contribute to the pathogenesis of postnatal mast cell disorders.

## Introduction

Mast cells (MCs) reside in mucosal and epithelial tissues throughout the body and exhibit various critical immune reactions, including allergies, infections, and tumor environment (1–5). However, the developmental origin of MCs has been a long-standing unresolved question (2, 6). While MCs can be induced from the adult bone marrow (BM) lineage negative Sca1^+^Kit^+^ (LSK) cells *in vitro* culture (7, 8), postnatal BM cells do not repopulate MCs upon transplantation (9). These contradictory results between *in vitro* and *in vivo* assays raised controversy regarding the origins of MCs. The only successful MC repopulation by BM transplantation used *Kit^W-sh^* mutant mice as recipients. *Kit^W-sh^* mutant mice lack MCs in the whole body without impairment of normal hematopoiesis (7, 9). Although adult BM transplantation into lethally irradiated *Kit^W-sh^* recipients can reconstitute MCs, it fails to regenerate MCs in non-irradiated *Kit^W-sh^* hosts (9), suggesting that both host MC depletion and irradiation are required for MC repopulation by adult BM progenitors (6, 7, 9). However, intrauterine transfer enables embryonic (E) day 12.5 (E12.5) fetal liver (FL) progenitors to repopulate MCs in the embryo without irradiation (10), suggesting that an optimal recipient environment is an important factor for MC repopulation.

Recent elegant fate-mapping studies revealed a dual embryonic origin of MCs (10, 11). In the mouse embryo, multiple waves of hematopoiesis arise from endothelial cells (ECs), including primitive erythropoiesis and microglia production at E7.5, definitive-type erythromyeloid progenitor (EMP) production at E8.5-9.5, and HSC emergence from hemogenic ECs at E10-11 (12, 13). Accordingly, the previous fate-mapping studies indicated that the early wave of MCs is derived from Cdh5^+^ ECs in the extraembryonic yolk sac (YS) possibly through EMP. Subsequently, the second wave of MCs is derived from the ECs in the aorta-gonad-mesonephros E10.5 (AGM) region (11) or E9.5 Runx1^+^ positive cells (10). However, *Cdh5*- or *Runx1*- fate mapping assays at 10.5 or E9.5 cannot limit HSC, instead, label all blood lineages (11, 14). Therefore, it remains unclear whether the second wave of MCs is produced directly from ECs or *via* the first HSCs produced in the AGM region. Further, it is not clear which hematopoietic wave contributes to the known MC progenitors (MCps), c-Kit^+^β7-integrin^+^ cells in the E12.5 FL (10).

In this study, we asked whether strictly defined fetal HSCs could give rise to MCs in the peritoneal cavity (PerC) and skin under physiological (fate-mapping) and transplantation settings. Using complementary EC- and HSC-fate mapping assays in addition to highly purified HSC transplantation into NOD/SCID/IL2γc^-/-^ (NSG) neonate, we show that the first emerging HSCs in the developing AGM region and E12.5 FL HSCs retain the potential to produce MCs. In clear contrast, FL HSCs at E14.5 and later including adult BM HSCs have almost no contribution to producing MCs in both transplantation and fate-mapping assays. Thus, our studies underpin the divergence of MC lineage and HSC in the early embryonic period and highlight the unique property of the first emerging HSC.

## Materials and Method

### Mice

C57BL/6 mice were used for timed pregnancy and transplantation donors for E11.5 to adult BM. Embryo ages were also confirmed by counting the somite numbers. C57BL/6, Boy/J, B6-Fgd5^ZsGreen-ERT2Cre^, B6.Rosa^CAG-LSL-tdTomato^ (Rosa^LSL-Tom^), Gt(ROSA)26Sort^m4(ACTB-tdTomato,-EGFP)Luo/J^ (Rosa^mT/mG^), B6.IgHa mice, and NOD.Cg-Prkdc^scid^IL2rg^tm1^ (NSG) mice were purchased from Jackson Laboratory. B6-Tg(Cdh5-cre/ERT2) Mice were purchased from Taconic (No:13073). Enhanced green fluorescent protein (pCx-eGFP) (15) and red fluorescent protein (TM7-RFP) (16) transgenic mice were kindly provided by the Weissman laboratory (Stanford). Runx1^Mer/Cre/Mer^ mutant mice (17) were provided by Riken Center for Life Science Technologies. All mice were maintained in the specific pathogen-free condition at the University of Texas Health Science Center at Houston or Emory University following the respective institutional IACUC protocols.

### Cell Preparation and Cell Sorting

Embryos were harvested and dissected in YS and AGM regions. Each tissue was dissected and digested in 0.25% collagenase (Stemcell Technologies) for 15–30 minutes at 37°C, followed by cell dissociation buffer (Life Technologies) to stop the reaction and make a single cell suspension. The fetal liver was dissociated and applied to mononuclear cell gradient centrifuge separation with Histopaque 1083 (Sigma) to obtain blood mononuclear cells (MNCs). Cell number was counted by trypan blue, and cells were stained with antibodies (Listed below) based on **Figure S1B**, followed by FACS sorting for purification with FACS Aria or FACS Melody (BD Biosciences). LT-HSCs were identified as lin^-^c-Kit^hi^Sca1^hi^(LSK)CD150^+^CD48^-^ (and, optionally, further CD41^-^, CD34^low^,CD45^+^, CD38^+^ and CD127^-^). As we did not use CD135 Ab, we defined adult BM MPP as LSKCD150^-^CD48^+^ cells, which is corresponding to MPP3/4 or MPP^Ly^+MPP^G/M^ elsewhere (18). FL MPP subpopulations have not been characterized well, therefore, LSKCD150^-^CD48^+^ cells or KSL CD201^-^(EPCR)^-^CD48^+^ cells were referred to as MPP in the FL (19). All antibodies were purchased from eBioscience, BD Bioscience, Tonbo Bioscience, or Biolegend.

### Transplantation

Day 1-3 NSG neonatal or adult mice (expressing CD45.1) were used as recipients and were irradiated (neonate: 150 or adult: 200 rad) before transplantation. Donor cells (CD45.2) were injected into the facial (neonate) or tail (adult) vein. Blood was taken periodically from the 4th week after transplantation and donor cell types were examined by flow cytometry (LSRII, BD Biosciences). Successful MC repopulation was defined as more than 0.1% donor-derived MCs among the total host MCs in PerC. Transplant experiments using RFP+ donor cells were performed as described (20, 21). Briefly, fetal livers from ~E15 TM7-RFP (RFP+) mice or BM from adult RFP+ mice (>20 wks) were stained with the mAbs listed below. Sorted LT-HSCs (~100 cells) from fetal liver or adult BM were injected intravenously (tail vein) into lethally irradiated (two doses of 4.25 Gy delivered 4 h apart) B6.IgHa mice along with ~2 x 10^5^ BM rescue cells from 8 wks old pCx-eGFP mice. In separate experiments, B6.IgHa mice received 1.5 x 10^6^ cells from unsorted fetal liver or adult BM. LT-HSCs were sorted on Emory Pediatric/Winship Flow Cytometry Core or Stanford Shared FACS Facility BD FACSAria II instruments.

### Fate-Mapping Study

Fgd5^ZsGreen-ERT2Cre :^ Rosa^LSL-Tom^ (iFgd5) and Cdh5^ERT2Cre :^ Rosa^LSL-Tom^ (iCdh5) mice were created by crossing Rosa^LSL-Tom^ with the respective Cre-bearing animals. Runx1^MER/Cre/MER^ and Rosa^mT/mG^ were crossed as described previously to create iRunx mice. Details of labeling were performed (20, 22), and Tamoxifen (TAM) was administrated on each pregnant day by oral gavage or by ip at p2 neonate. For the iRunx experiments, 4-hydroxytamoxifen (4-OHT) and progesterone were administered by ip injection to the pregnant dam at E8, as reported (20). After delivery, we harvested multiple organs and analyzed tdTomato (iCdh5 and iFgd5) or eGPF (iRunx) percentage in every target population.

### Antibodies

The antibodies used include: anti-CD45.2 (clone 104), CD45.1 (A20), CD19 (1D3), CD4 (GK1.5), CD8 (53-6.7), CD3 (145-2C11), IgM (RMM-1), CD5 (53-7.3), CD11b (M1/70), Gr-1 (RB6-8C5). CD144 (clone:11D4), CD45 (30-F11), NK1.1 (PK136), CD41 (MWReg.30), c-Kit (2B8), Ter119 (TER-119), CD201 (1560), Sca-1 (D7), CD48 (HM48-1), CD150 (TC15-12F12.2), β7-integrin (FIB27), FcERI (MAR-1), F4/80 (BM8), CD127 (A7R34).

## Results

### Efficient MC Repopulation in Neonatal NSG Recipient Mice

**Figure 1:**
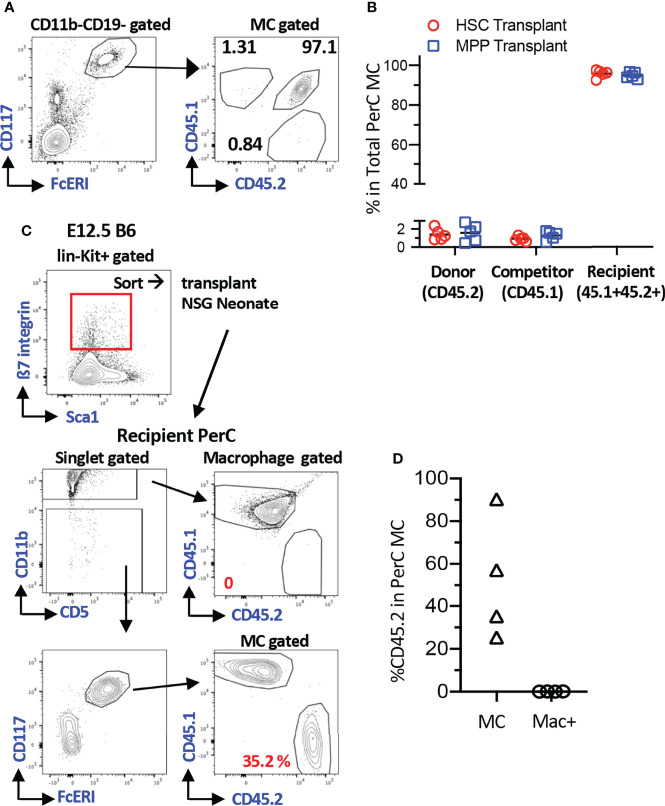
Efficient MC repopulation in neonatal NSG recipient mice. **(A)** Representative flow plots of recipient’s PerC cells transplanted with BM HSC cells. Sorted 100 adult BM HSCs or 3000 MPPs (B6; CD45.2) were transplanted into irradiated F1 B6/BoyJ (CD45.1+CD45.2+) mice together with 3x10^5^ BoyJ BM MNCs (CD45.1). **(B)** Percentages of the donor, competitor, and recipient MC chimerism in the recipient’s PerC. (n=5 each) **(C)** A gating strategy for sorting of c-Kit^+^β7-integrin^+^ MC progenitor cells from E12.5 B6 FL (Top Panel). Representative gating for MC and macrophage populations in PerC of recipient NSG mice 2 months after transplant with 3500 of Kit^+^β7-integrin^+^ cells (bottom panel). **(D)** % reconstitution of MCs and Macrophages population in PerC of recipient NSG mice as in C(n=4).

To understand the MC producing capacity of HSCs at various ages, we sought the most optimal recipients that can yield donor-derived MCs. To quantify the donor-derived MC repopulation, we focused on the PerC MCs because they were easily harvested, and precise numerical evaluation can be applied using Flowcytometry. First, we tested MC capacity of adult BM HSCs in the conventional method. LT-HSCs (CD150^+^CD48^-^ LSK cells) or multipotent progenitors (MPP3/4s, CD150^-^CD48^+^ LSK) were sorted from CD45.2^+^ C57BL/6 mice and transplanted into lethally irradiated F1 mice (CD45.2^+^CD45.1^+^) with competitor cells (CD45.1^+^), and MCs engraftment in the recipient PerC was examined. As previously reported, we confirmed the lack of MC repopulation (**Figures S1A, B** for gating strategy of MCs) from the donor and competitor cells (**Figures 1A, B**). Although *Kit^W-sh^* mutant mice are the best recipient mice for the MC engraftment assay, we sought alternative recipients to evaluate MC repopulation capacity. It has been reported that c-Kit^+^β7-integrin^+^ cells from E12.5 FL repopulated MCs in C57BL/6 embryos *via in-utero* transplantation (10). Therefore, c-Kit^+^β7-integrin^+^ cells from E12.5 FL are the only known MC progenitors that can repopulate regular C57BL/6 mice (although they were embryos). Based on this, we decided to evaluate the feasibility of neonatal NSG recipients for the MC repopulation assay, by transplanting E12.5 FL c-Kit^+^β7-integrin^+^ cells into NSG neonates instead of *in-utero* transplantation because NSG neonates still maintain the fetal environment that accepts immature fetal cells for the physiological hemato-immunological development compared to adult recipients (23–25). We sorted 3500 lin^-^c-Kit^+^β7-integrin^+^ cells from E12.5 FL (**Figure 1C**; top panel) and transplanted them into NSG neonates after 150 cGy irradiation. Two months after transplantation, MCs, but not macrophages or other lineage cells, were efficiently repopulated (**Figure 1D**). Since the least preconditioning to NSG neonate would not eradicate host MCs, which was considered to be necessary (9), we measured the remaining MC counts in the PerC after irradiation. We irradiated adult and neonatal NSG mice and determined PerC MC numbers 3-4 months after irradiation. We found that MCs in the PerC in both adult and neonatal NSGs remained, with a comparable number to that of non-irradiated NSG or control C57BL/6 (**Figure S1D**). As *in-utero* C57BL/6 embryo can accept MC engraftment without preconditioning, the fetal and neonatal environment seems to be an important factor for MC engraftment. These results demonstrated that NSG neonates are valid recipients for MC repopulation assays despite abundant host MCs.

### AGM Pre-HSC and FL HSC at E12.5 Possess MC Repopulating Capacity, but Not After E14.5

**Figure 2:**
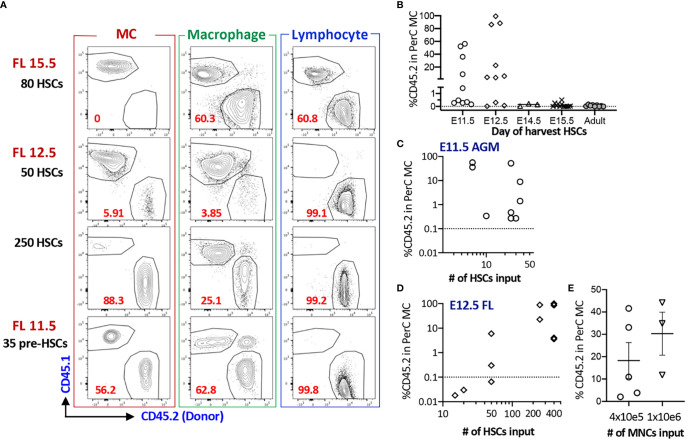
AGM pre-HSC and FL HSC at E12.5 possess MC repopulating capacity, but not after E14.5. **(A)** Representative flow plots of MC, macrophage, and lymphoid reconstitutions from 15.5 FL, 12.5 FL HSC or E11.5 AGM pre-HSCs in PerC (gating strategy provided in S1B). **(B)** % Donor (CD45.2) contributions of MCs in PerC based on donor ages. (n=32 for 11.5, n=27 for E12.5, n=4 for E14.5, n=11 for E15.5, n=9 for adult BM). **(C, D)** Numbers of HSC transplanted and % donor (CD45.2) in the total host PerC MCs are shown for E11.5 AGM pre-HSCs **(C)** and E12.5 FL HSCs **(D)**, **(E)** Repopulation of MCs in PerC of NSG recipients transplanted with E14.5 FL donor MNCs are shown (n=5 and 3 in independent experiment respectively).

Next, we assessed whether fetal HSCs have MC repopulating capacity because previous studies investigated only adult BM cells. When E15.5 FL LT-HSCs were transplanted into lethally irradiated adult mice, they failed to repopulate PerC MCs despite complete multi-lineage chimerism in the host (**Figures S2A–C**, **Table S1**). Therefore, we decided to use NSG neonates as recipient mice thereafter. Although E14.5 FL HSCs failed to repopulate PerC MCs, FL mononuclear cells (MNCs) successfully repopulated MCs, indicating the presence of MCps that must be produced in the earlier embryonic stage. Therefore, we examined MC capacity of HSCs in earlier stages. We sorted E12.5 FL HSCs (**Figure S1C** for gating strategy) and transplanted various numbers (5 to 250) HSCs into NSG neonates. In contrast to E15.5 FL HSCs, E12.5 HSCs showed successful MC repopulation (**Figures 2A, D**) while E12.5 MPPs showed MC repopulation only in one-third of recipient mice with lower chimerism (**Table S1**). Therefore, we examined the MC capacity of the earliest HSCs or HSC-precursors (pre-HSCs) that can be detected in the E11.5 AGM region (26). Pre-HSCs are intermediate precursors between hemogenic ECs and HSCs and express CD144^+^(VE-cadherin^+^) c-Kit^+^EPCR^++^ (**Figure S1C**). We have previously reported that highly purified EPCR^bright^ pre-HSCs can reconstitute multi-lineage blood cells in neonatal NSG hosts (24). Therefore, we transplanted 35 pre-HSCs into sublethally irradiated NSG neonates and found a robust MC repopulation (**Figures 2A, C**). Surprisingly, as few as six sorted pre-HSCs showed remarkable MC repopulation in PerC with multi-lineage repopulation (**Figure 2C**), indicating that E11.5 HSCs have a strong MC repopulating capacity. When we compared the numbers of injected HSCs from E11.5 AGM or E12.5 FL and repopulated MC %, we saw that MC repopulating capacity was declining from E11.5 AGM to E12.5 FL HSCs (**Figures 2C, D**).

Taken together, fetal HSCs possess MC repopulating capacity between E11.5 to 12.5, but not thereafter.

### E12.5 FL Fgd5+ Cells Consist of HSCs and Precursors With MC Potential

**Figure 3:**
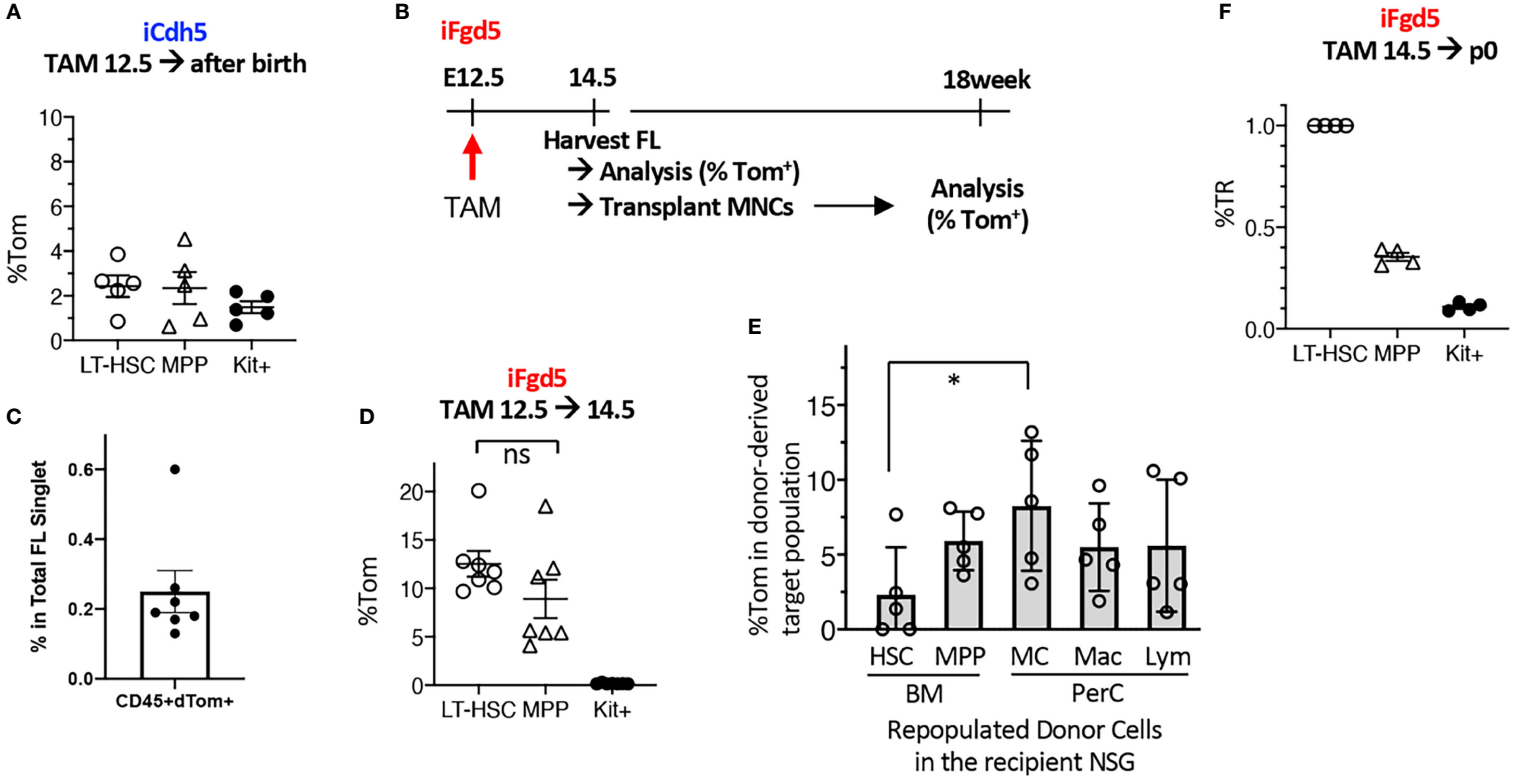
E12.5 FL HSCs are not exclusively labeled by iCdh5 nor iFgd5. **(A)** TAM injections into iCdh5 embryos at E12.5 were performed and %Tom was measured in various immune lineages after birth (n=5). **(B)** Experimental timeline is shown. **(C)** TAM was injected into iFgd5 mice at E12.5 and analyzed at E14.5 FL. %Tom in LT-HSC, MPP, and Kit^+^ are shown (n=7). **(D)** TAM was injected at E12.5 into iFgd5 embryo and analyzed FL at E14.5, then 4x10^5^ FL MNCs from each cre (+) embryo were transplanted into adult NSG recipients, followed by lineage analysis at 18 weeks after transplant. %Tom of various fractions from recipients (post-transplant donor cells) are shown (*: p<0.05, n=5). **(E)** TAM E14.5 into iFgd5 was given and measured %TR at p0 (n=4). **(F)** %TR of LT-HSC, MPP, and c-Kit+ cells in the P0 neonatal BM when TAM was injected at E14.5. ns, not significant.

Based on the successful MC repopulation using E11.5 AGM pre-HSC and E12.5 FL HSCs, we tried to label E12.5 FL HSCs using fate-mapping mouse models. We used complementary fate-mapping models to label both Cdh5^+^ endothelial cells (ECs) and Fgd5^+^ HSCs, using iCdh5 (Cdh5^ERT2Cre :^ Rosa^LSL-Tom^) and iFgd5 (Fgd5^ZsGreen-ERT2Cre :^ Rosa^LSL-Tom^) mice, respectively. Cdh5 encodes VE-cadherin, specifically expressed in the ECs, and Fgd5 is exclusively expressed in HSCs (27, 28). Because the tdTomato (Tom)-labeling efficiency is a variable, we always calculated the percent tdTomato ratio (%TR), measured by the ratio of %Tom^+^ of a defined cell population divided by the %Tom^+^ of HSCs in the same host (29). Tamoxifen (TAM) injection into E12.5 iCdh5 (EC fate mapping) resulted in only up to 4% labeling efficiency in BM HSC/MPPs after birth (**Figure 3A**) despite the clear expression of CD144 (VE-cadherin) in the E12.5 HSC population (**Figure S3A**). This suggests that the Cdh5 transcript is already downregulated in the E12.5 HSCs. Therefore, we switched to iFgd5 mice (HSC fate mapping) because the iFgd5 mouse is an established model for labeling adult BM HSCs (29, 30). We confirmed that the ZsGreen reporter (indication of Fgd5 expression of the Fgd5^ZsGreen-ERT2Cre^ mouse) showed the brightest signal in the E12.5 FL HSC compartment defined by CD48^-^EPCR^++^LSK, but also dim positive signal in CD48^+^EPCR^-^ MPP population (**Figure S3A**). When we injected TAM into iFdg5 at E12.5, followed by the analysis of FL at E14.5, we found that %Tom^+^ was only 0.25% in the total CD45^+^ cells, but exclusively enriched in the LSK population (**Figures 3C**, **S3B**). In the LSK cells, both HSCs and MPPs labeled with Tom were at comparable levels (**Figure 3D**). Accordingly, Tom^+^ cells labeled at E12.5 were composed of HSC, MPP, and progenitors in E14.5 FL (**Figures S3B, S3C**). These results showed that TAM injection into iFgd5 at E12.5 labeled both HSC and MPP together.

As E14.5 FL MNCs, but not FL LT-HSCs, repopulated MCs (**Figure 2E**), we sought the origin of MCs in the E14.5 iFgd5 FL MNCs in which HSCs and MPPs had been labeled at E12.5. **Figure 3B** shows the experimental timeline. We transplanted E14.5 FL MNC from iFgd5 embryos labeled at E12.5 into NSG recipient mice and examined Tom% between donor-derived HSCs and MC in the recipient mice 18 months after transplantation. The percentage of Tom^+^ in donor-derived PerC MCs was significantly higher than that in donor-derived LT-HSCs in the BM of the recipient mice 18 weeks after transplantation (**Figure 3E**, *p<0.05, n=5). Further, some recipient mice showed higher Tom% in MCs than that in MPPs in the recipient BM, suggesting the presence of MC precursors other than MPPs. When TAM was injected into E14.5 iFgd5 embryos, HSCs were exclusively labeled while MPPs and c-Kit^+^ cells were less labeled when analyzed at P0 (**Figure 3F**), suggesting that E14.5 TAM injection labeled LT-HSCs more specifically. Lastly, a single injection of TAM at p2 remarkably labeled the HSCs compared to the single injection of TAM at E14.5 (**Figure S3D**). Taken together, these results demonstrate that E12.5 FL Fgd5+ cells include HSCs and various precursors with MC potential, and selective HSC-labeling in the iFgd5 model is available after E14.5.

### Fate-Mapping Assays Reveal an HSC-Independent, but EC-Dependent Origin for MCs

**Figure 4:**
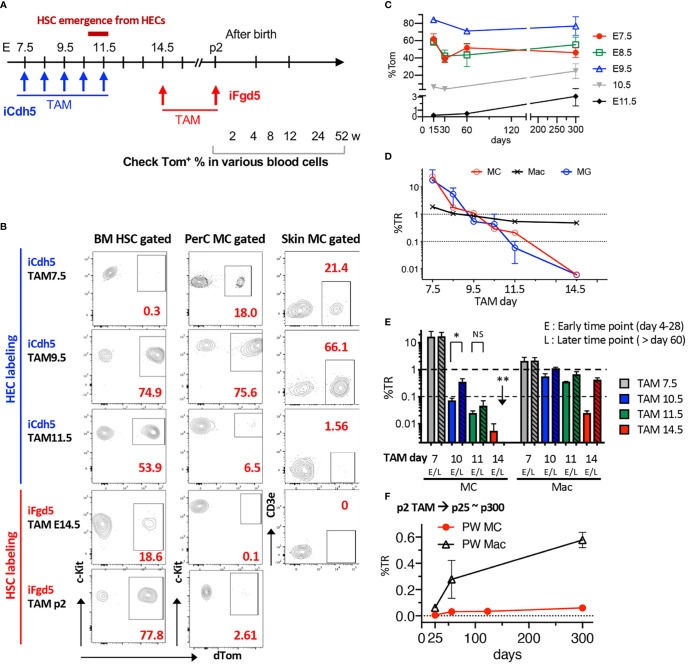
Fate-mapping assays reveal an HSC-independent, but EC-dependent, origin for MCs. **(A)** Time points of TAM injection into iCdh5 or iFgd5 mice and various time points for analysis are depicted. **(B)** Representative flow plots for %Tom in BM HSCs, PerC, and skin MCs based on each TAM injection day (n=2-5 in each point). **(C)** Absolute percentages of Tom in PerC MCs were measured in iCdh5 mice. %Tom in various time points of analysis based on each embryonic day of TAM injection are plotted (n=3-7 in each point). **(D)** %TR of PerC MC, Macrophage (Mac), and Brain MG based on multiple TAM time points are plotted (n= 3-6 in each time point). **(E)** Kinetics of %TR of MC and Mac populations in PerC between early (<28 days) and late time points (>60 days) based on TAM injection day (n= 3-7 in each point). *: p<0.001(n=4). **: p<0.001 (n=3). **(F)** TAM was injected into p2 iFgd5 neonate and %TR of MC and Mac populations in PerC were plotted until one-year-old (n=3 in each point). ns, not significant.

Once confirmed that TAM injection into iFgd5 mice at E14.5 efficiently labeled FL HSCs, we labeled HSCs in E14.5 FL and p2 BM and traced HSC-derived blood cells at various time points to confirm no MC production from HSCs in a physiological setting. We also traced EC-derived MCs by using iCdh5 mice to seek the MC origin (**Figure 4A**). Representative FACS plots of BM HSCs and PerC MCs labeled at various time points are shown in **Figure 4B**. In EC fate-mapping mice (iCdh5), TAM injection at E7.5 displayed greater percentages of Tom^+^ cells in both PerC and skin than that in BM HSCs. This HSC-independent MC production was also confirmed using Runx1^Mer/Cre/Mer :^ Rosa^mT/mG^ (iRunx1) mice. TAM injection into iRunx1 mice at E8.0 marked only MCs in adult mice without labeling other lineages in PerC (**Figure S4**). When TAM was injected into iCdh5 mice at E9.5, an equivalent labeling efficiency between MC and HSC was observed in adult mice (**Figure 4B**), indicating that E9.5 ECs produce both MCs and HSCs. TAM injection at E11.5 showed a global reduction in labeling efficiency, but relatively higher labeling was observed in HSCs while MC labeling was markedly reduced (**Figure 4B**, middle). In contrast, TAM injection in iFgd5 mice (HSC-labeling) at E14.5 and P2 showed very poor labeling in the MC compartment (**Figure 4B**, bottom). **Figure 4C** shows the absolute percentage of Tom^+^ in PerC MC at various time points after birth based on each TAM injection day into iCdh5 mice. E9.5 TAM achieved the highest recombination efficiency in both early and late time points than that on other days. E7.5 TAM showed the trend of decreasing the %Tom in later time points, in contrast, E10.5 and 11.5 TAM resulted in a lower Tom^+^ percentage in the early time point, then increased Tom positivity at the one-year time point that was in line with the previous report (11) and our transplant results (**Figures 2C, D**). The kinetics of %TR of PerC MCs for each TAM injection time point was calculated and plotted alongside the kinetics of brain microglia (MG), which are also known to develop through an HSC-independent pathway (**Figure 4D**) (20, 30). The %TR of MC progressively decreased with fetal age, well overlapping with the kinetics of brain MG. At the same time, PerC macrophages which are known to develop from LT-HSCs (20) kept %TR close to 1.0, likely reflecting their HSC origin. In addition to the kinetics of absolute %Tom in **Figure 4C**, we compared %TR of MC and macrophages at early or late time points after birth when TAM was injected in iCdh5 mice at E7.5, E10.5, or E11.5, or in iFgd5 mice at E14.5 (**Figure 4E**). Compared to the high %TR in MCs at E7.5 TAM injection, TAM injection at E10.5 and E11.5 showed lower %TR in MCs at the early time points (within p28) and subsequently gradual increase in their contribution, being consistent with the previous reports (11). However, when TAM was injected into iFgd5 mice at E14.5, the %TR in MCs was very low and diminished by the later time point (**Figure 4D**). In contrast, %TR in macrophages exhibited quite low at an earlier time point but subsequently increased closer to 1, indicating HSC-dependent generation of macrophages. When TAM was injected at P2, PerC MCs Tom labeling kept very low even after one-year post-TAM injection (**Figure 4F**, %TR = 0.049 ± 0.03, n=3). This data suggests that more than 95% of PerC MCs were generated and maintained by prenatally produced cells while macrophages reached %TR near 0.7. (**Figure 4F**). These results show that FL HSCs at E14.5 and later have the least contribution to PerC MC production, which is in line with our FL HSC transplantation results (**Figures 2B**, **S3**). Thus, most MCs are produced and maintained prenatally in a physiological setting, independently of the HSCs.

## Discussion

In this study, we combined several *in vivo* fate-mapping mouse models and transplantation assays and demonstrated that FL HSCs have a potential to generate MCs in a limited time window. The first emerging HSCs can regenerate MCs only between E11.5 to E12.5, while most MCs are produced by prenatal HSC-independent progenitors, including ECs from E7.5 to E10.5. Our data provided compelling evidence for an HSC-independent origin for MCs and a divergence between HSC and MC lineages in the developing embryo.

Originally, MCs were believed to represent a mesenchymal lineage since its discovery by Paul Ehrlich more than 100 years ago (2, 6). Kitamura’s group first challenged this theory about 40 years ago, providing evidence that MCs emerge from HSCs. Since then, the notion that MCs originate from HSCs has become the accepted paradigm in the field (31–34). Upon discovering MC progenitors in the adult BM (35, 36), it has been speculated that HSC-derived MC progenitors leave the BM, circulate in the blood, and distribute across peripheral tissues to complete terminal differentiation. Recent progress in developmental immunology has again challenged the current paradigm and demonstrated that many tissue-resident immune cells develop through an HSC-independent pathway, including direct development from hemogenic ECs (11, 13, 20, 30, 37–39).

Our previous report with detailed EC fate mapping and transplant studies revealed that the frequency of the functional HSCs at E10.5-E11.5 AGM is very low (<1 cell/embryo) (22, 24), and the majority of iCdh5+ or Runx1+ (14) cells in the AGM region at that time are MPPs/progenitors, rather than HSC. Therefore, to address the potential of HSCs to regenerate MCs, we needed to employ a complementary HSC fate mapping mouse model. However, finding a specific marking of HSC is also challenging. Currently, the iFgd5 is thought to be the best model regarding specificity and recombination efficiency compared to Tie2, Pdzk1ip1, or KRT18 (19, 27, 40–42). Additionally, intrauterine labeling is less efficient than postnatal p2 marking as **Figure S3D**. It is thought that lower recombination efficiency potentially influences the results (43). Nonetheless, iFgd5 mice showed that TAM injection at P2 labeled 70-100% of BM HSCs and still did not label MCs for life. This is the first fate-mapping model demonstrating the limited MC production ability of HSCs in a physiological setting. Further, despite the limitation in efficacy and specificity of HSC labeling in the embryonic period during HSC emergence, HSC transplantation assays can provide complementary information regarding the MC regenerative potential. In our HSC transplantation assays, we demonstrate that the earliest multi-lineage reconstituting HSCs found at E11.5 AGM have the potential to regenerate MCs in NSG recipients.

One of our intriguing findings is that host MC eradication is not required to detect the MC potential of donor cells. As **Figure S1D** shows, irradiated NSG neonates and adults remain abundant numbers of MCs in PerC, but, nonetheless, we observed significant host MCs after FL progenitors repopulated MCs (**Figure 1C**; **Table S1**). On the contrary, one example of the adult C57/BL6 recipients showed no FL-HSC-derived MC reconstitution despite the disappearance of the host MC after irradiation, indicating that diminishment of MCs did not help donor-MC engraftment (**Figure S2E**). Thus, our result suggests that intrinsic MC potential in precursors, which is developmentally characterized, is a more important determinant factor than the host environment to achieve MC reconstitution. However, the sensitivity of recipient mice that yield donor-derived MCs is also a critical factor to investigate MC potential in fetal progenitors. It has been reported that matching the developmental stages of donor cells and recipient mice resulted in better donor cell engraftments (44). NSG neonates are permissive to embryo-derived cell engraftment compared to adult recipient mice and showed better donor-derived engraftment by direct injection of early embryonic progenitors (24–26, 45, 46). Because MCs are derived only from the early embryonic progenitors before E12, our transplantation system using NSG neonates enabled us to detect their limited potential, which would not be detected by using wild-type adult recipient mice.

**Figure 5:**
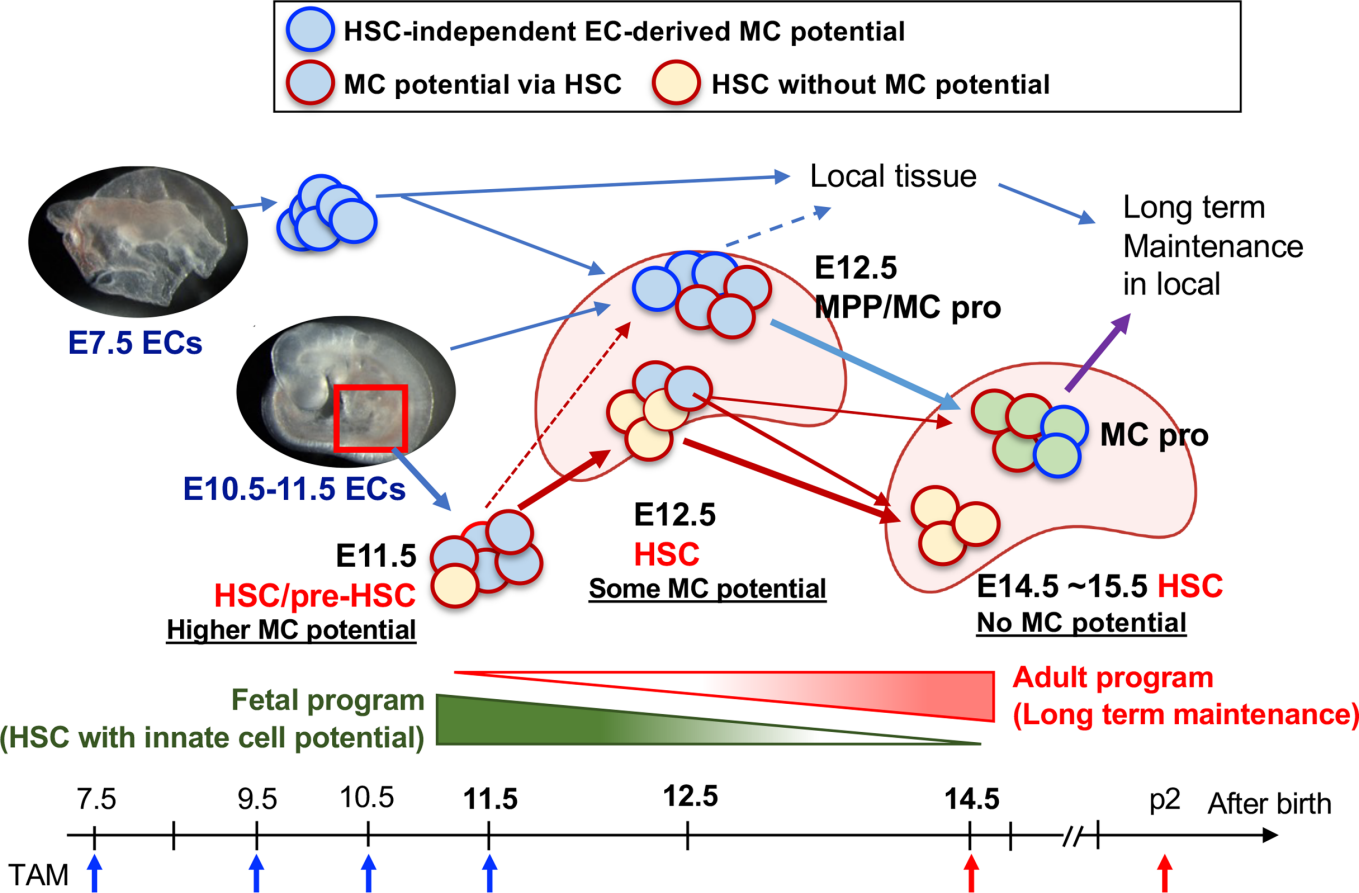
Diversification of HSC to the MC progenitor and HSC maturation. E10.5–11.5 pre-HSC in the AGM region contains a variety of cell populations including HSC with MC capacity (plus other innate lineages too), MPP, and other lineage-restricted progenitors. E12.5 FL contains at least 3 classes of MC repopulating cells; 1) HSCs (with reduced MC differentiation capacity), 2) MPPs with MC potential, and 3) Kit^+^β7-integrin^+^ MC progenitor cells. 1) is the consequence of E11.5 pre-HSC/HSC and 2) are derived from E9.5-10.5 AGM EC-derived production. 3) are derived from the early EC-derived wave and later AGM EC wave. In E14.5, HSCs had lost MC capacity, but MC progenitors still exist within MNCs, then distribute into general tissue and maintain long term in local.

In addition to the previous results describing “definitive MC-poiesis” by iCdh5 with E10.5 labeling (11), we clarified the contribution of pre-HSCs and HSCs to the PerC MCs. With our data and other studies, we propose MC development as depicted in **Figure 5**. The first early wave of MC progenitors is produced from ECs at E7.5, which migrate to the FL at E12.5 as well as relocating to local tissue, while E10.5-11.5 EC-derived pre-HSCs also possess MC potential, which also seed the FL. This MC potential is transiently retained in the FL HSC until E12.5 and is lost before the E14.5 stage. Hematopoietic progenitors possessing MC potential in the E14.5 FL include MPPs and β7-integrin^+^ MCps. In our data, whether β7-integrin^+^ MCps in the FL are derived through EMP or HSCs remains unclear. E10.5-11.5 AGM contains a variety of HSC-independent progenitors capable of regenerating innate and adaptive immune cells, including macrophages, MCs, B-1 B-cells (24), and αβ T-cells (47). Moreover, our transplant data showed the presence of multilineage repopulating HSCs with a great MC and B-1a cell reconstitution ability (22, 24). Thus, it is assumed that the earliest HSCs emerging from ECs in the AGM retain the program to generate multiple tissue-resident innate immune cell lineages, but they lose this innate program during undergoing HSC maturation in the FL, acquiring self-renewal/long-term repopulating capacity as definitive HSCs by E15.5, (**Figure 5**). The mechanism through which this dramatic change of HSC capacity occurs remains unknown and needs to be elucidated.

Another important question is the relationship between HSC, MPP, and β7-integrin^+^ MCp in E12.5 FL. As shown in **Figure 1C**, a certain percentage of Kit^+^β7-integrin^+^ cells in the E12.5 FL are Sca1^dim^ positive, therefore the MPP that showed MC repopulation ability in **Table S1** might have included β7-integrin^+^ MCps. MC repopulation ability in E10.5 pre-HSCs and E12.5 β7-integrin^+^ MCp can be detected only when the recipients are embryos or NSG neonates (24), suggesting that MC repopulation potential requires a special environment. These data display the limitation of transplantation assays. Since E10.5-12.5 is a critical diversification time point of MC or HSC differentiation, a better fate-mapping model or *in-vivo* barcoding mouse model combined with single-cell RNA-seq and/or ATAC-seq will provide insights into the developmental pathways to address this intriguing question.

## Data Availability Statement

The original contributions presented in the study are included in the article/**Supplementary Material**. Further inquiries can be directed to the corresponding authors.

## Ethics Statement

The animal study was reviewed and approved by University of Texas Health Science Center at Houston, Animal Welfare Committee and by Emory University Institutional Animal Care and Use Committee.

## Author Contributions

MK, EG, and MY conceived, designed, and performed experiments and analyzed the results, wrote, and edited the manuscript. AK, SC, NV, HC, AL, and CN performed experiments. All authors contributed to the article and approved the submitted version.

## Funding

This work is partially supported by NIH R01AI121197 (MY), NIH R01AI123126 (EG), and Lowance Center for Human Immunology (EG).

## Conflict of Interest

The authors declare that the research was conducted in the absence of any commercial or financial relationships that could be construed as a potential conflict of interest.

## Publisher’s Note

All claims expressed in this article are solely those of the authors and do not necessarily represent those of their affiliated organizations, or those of the publisher, the editors and the reviewers. Any product that may be evaluated in this article, or claim that may be made by its manufacturer, is not guaranteed or endorsed by the publisher.
