# Supplementary material for: Mast Cell Repopulating Ability Is Lost During the Transition From Pre-HSC to FL HSC
Source: Front Immunol. 2022 Jul 8;13:896396. doi: 10.3389/fimmu.2022.896396 (PMC9309215; doi:10.3389/fimmu.2022.896396)
Supplement: Supplementary file 1 [file DataSheet_1.pdf]

Fig. S1

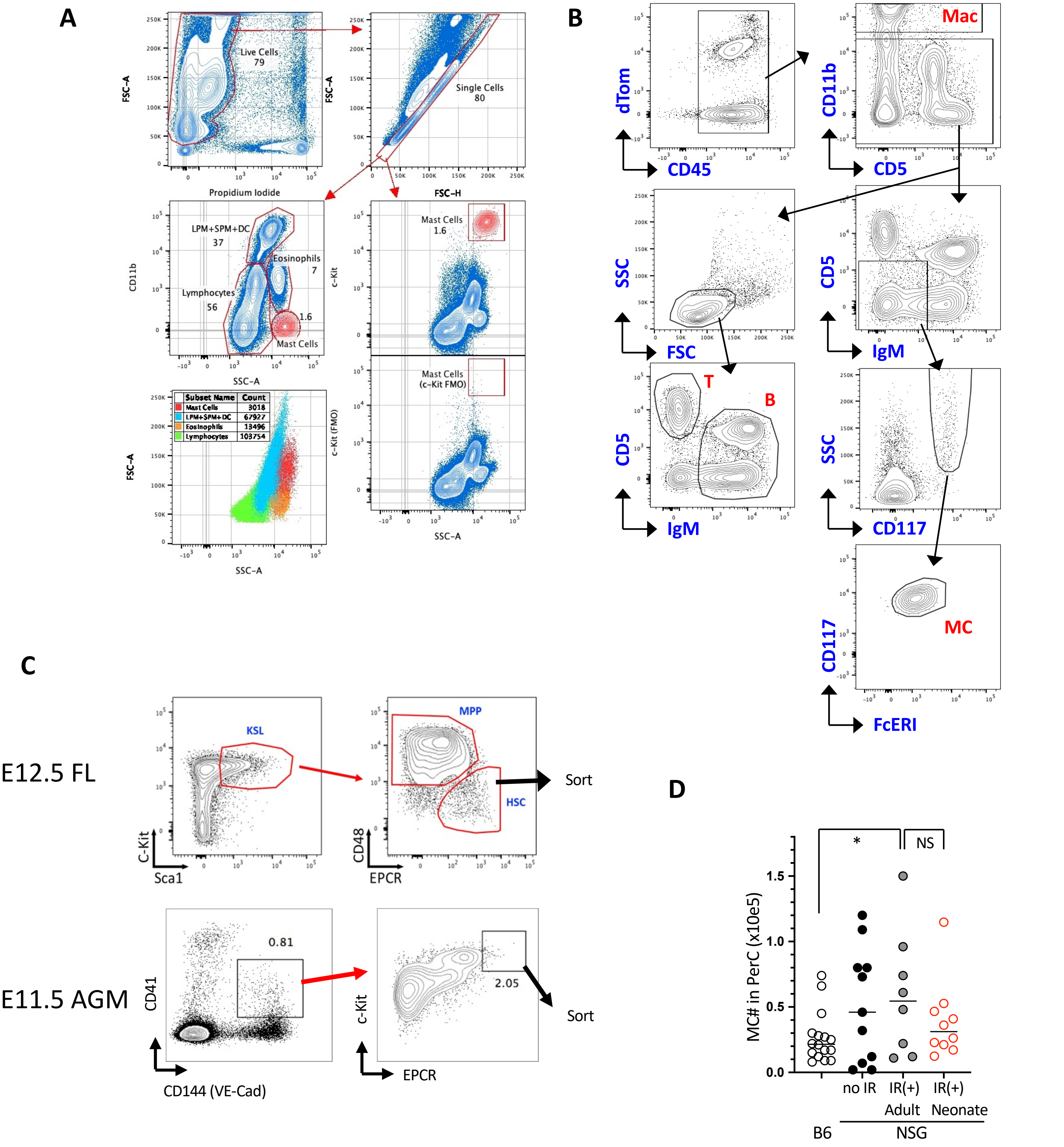

Fig. S1

A. Representative flow plot for the gating strategy of MC in PerC. B. Representative gating strategy for each lineage cell including MC in PerC. C. Gating strategy for E12.5 HSC and E11.5 AGM pre-HSC sorting. D. MC counts in PerC of control adult B6s, non-irradiated (no IR) adult NSGs, and NSGs irradiated (IR) at adult aged (4-6 months old) and adult NSGs irradiated at neonate (p1-3) are determined and plotted. N=8-17, *$p < 0.03$

Fig. S2

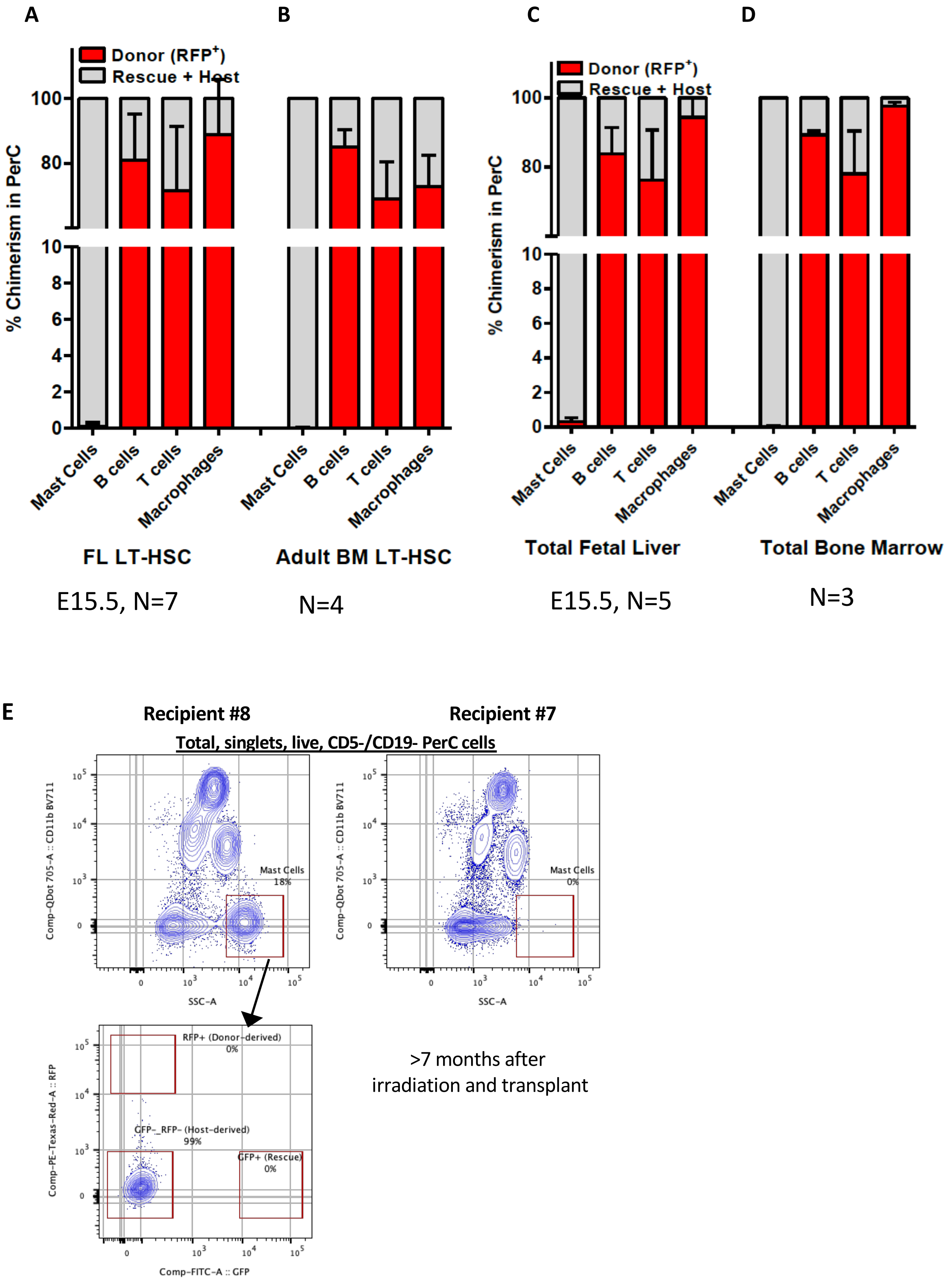

Fig. 2S

A - D. Representative results showing one series of MC repopulation assay. FL LT-HSC (A), Adult BM LT-HSC (B), FL MNCs (C) or Adult BM MNCs (D) from RFP+ mice were transplanted into B6.IgHa recipients. Rescue and host cells were injected together. MC and other lineages in PerC were analyzed and % chimerism were graphed. E. Representative case of FL HSC transplant showing diminishment of PerC MC. Nearly 8 months after transplant, recipient #7 showed no donor-derived MC repopulation as well as depletion of host-derived MCs. Recipient #8 contained recipient-derived MC in post-transplant 8 months.

Fig. S3

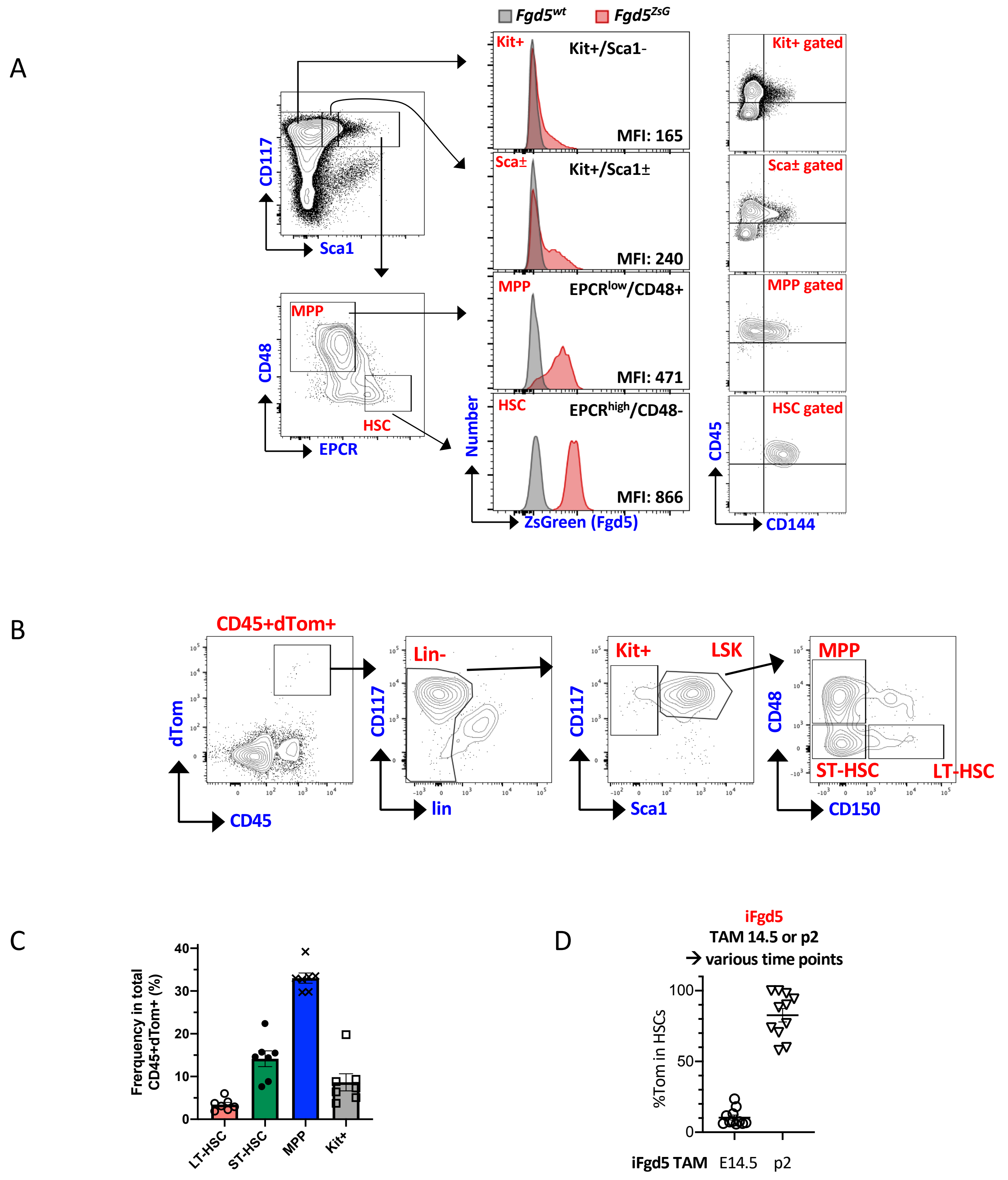

Fig. S3

A. Expression of Fgd5^{ZsG} reporter and CD144 in FL at E12.5. HSC, MPP, and c-Kit^{+} cells are gated and Fgd5 reporter and CD144 are shown. B. Representative dot plot showing population distribution of gated CD45^{+}Tom^{+} cells. E14.5 iFgd5 FLs, with TAM injection at E12.5, were harvested and analyzed. C. The frequency of targeted compartment (LT-HSC, ST-HSC, MPP, or Kit+) in the total CD45+dTom+ cells is graphed (n=7). D. Absolute percentages of Tom^{+} in the HSCs by the TAM at E14.5 or p2 are shown.

Fig. S4

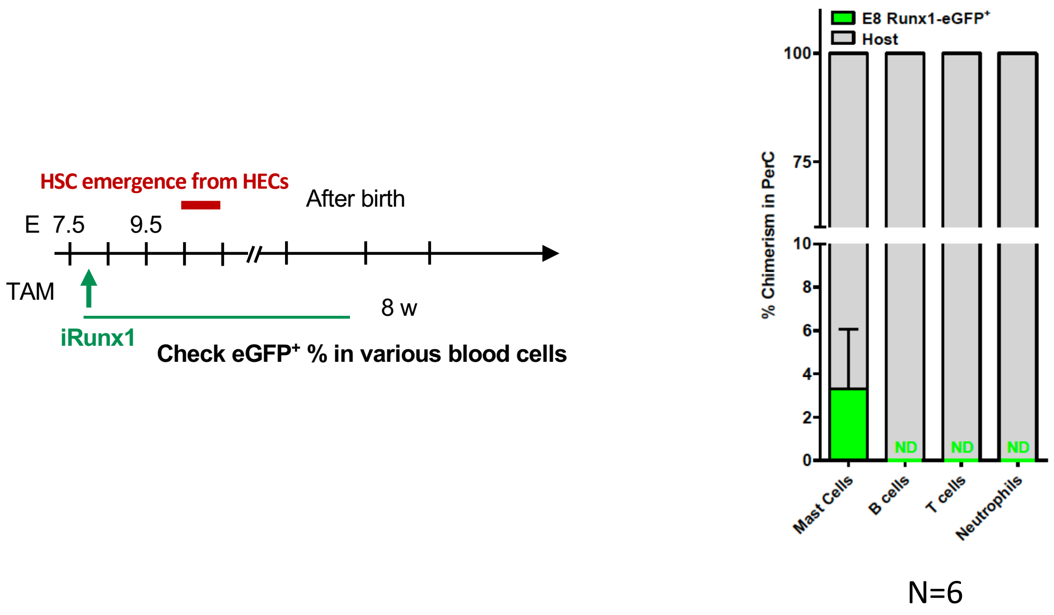

Fig. S4
iRunx1 marking result. TAM was injected at E8.0 and %eGFP positivity in various lineages in PerC were measured 8 weeks after delivery (n=6).

Table 1. Summary of in-vivo MC repopulation after transplantations

Donor		#	Recipient	XR(rad)	Competitor	n	**MC repopulation**	
**Adult**	LT-HSC	100	F1	900	(+)	9	(-)	
	MPP	3000	F1	900	(+)	5	(-)	
	Whole BM	1.5 M	B6.IgHa	850	(+)	3	(-)	
**E15.5**	FL-HSC	80	B/J	900	(+)	11	(-)	
	FL-MNC	1.5 M	B6.IgHa	850	(-)	5	(±)	
**E14.5**	FL-HSC	20	B/J	900	(+)	5	(-)	
	FL MNC	400K	NSG (Ad)	200	(-)	5	5 / 5	
	FL-MNC	1 M	NSG (Ad)	200	(-)	3	3 / 3	
**E12.5**	FL-HSC	5-50	NSG (Neo, Ad)	150	(-)	21	2 / 16*	(neo: 1/ Ad: 1)
	FL-HSC	80-400	NSG (Neo, Ad)	150	(-)	15	6 / 15	(Neo: 2/ Ad: 4)
	FL-HSC	2500	NSG (Neo, Ad)	150	(-)	3	3 / 3	(neo: 2 / Ad: 1)
	MPP	1200-2200	NSG (Neo, Ad)	150	(-)	6	2 / 6	(Neo: 1/ Ad: 1)
	β7 Integrin^{+}	3500	NSG (Neo)	150	(-)	4	4 / 4	(Neo: 4/ Ad: 0)
**E11.5**	Pre-HSC	6-40	NSG (Neo, Ad)	< 250	(-)	32	4 /22*	(Neo:3/ Ad: 1)

*: 5/21 in E12.5 (5-50 cells) and 10/32 in E11.5 showed no donor cell repopulation

Neo: Neonate, Ad: Adult
